# Dressed for the Weather: Tawny Owl Feather Adaptations Across a Climatic Gradient

**DOI:** 10.1002/ece3.71441

**Published:** 2025-06-24

**Authors:** Charlotte Perrault, Miguel Baltazar‐Soares, Chiara Morosinotto, Patrik Karell, Karel Poprach, Lars‐Ove Nilsson, Daniel Eriksson, Peter Ericsson, Gintarė Grašytė, Saulius Rumbutis, Daniele Baroni, Katy Anderson, Ingar Øien, Maria Casero, Jon E. Brommer

**Affiliations:** ^1^ Department of Biology University of Turku Turku Finland; ^2^ Department of Bioeconomy Novia University of Applied Sciences Finland; ^3^ Department of Biology University of Padova Padova Italy; ^4^ National Biodiversity Future Center (NBFC) Palermo Italy; ^5^ Department of Biology, Evolutionary Ecology Unit Lund University Lund Sweden; ^6^ Department of Ecology and Genetics University of Uppsala Uppsala Sweden; ^7^ TYTO, z. s. Věrovany Czech Republic; ^8^ Faculty of Science Palacky University Olomouc Czech Republic; ^9^ Karlsborg Sweden; ^10^ Fagersanna Sweden; ^11^ Januliškis Lithuania; ^12^ Nature Research Centre Vilnius Lithuania; ^13^ ISPRA ‐ Italian Institute for Environmental Protection and Research Bologna Italy; ^14^ Environment Forester Forestry and Land Scotland Aberfoyle UK; ^15^ BirdLife Norway Trondheim Norway; ^16^ Wildlife Rehabilitation and Research Center – RIAS Olhão Portugal

## Abstract

Populations are presumed to be adapted to local environmental conditions via natural selection, with gene flow breaking up local adaptations. In birds, various aspects of feathers may reflect local adaptation. For example, the insulation capacity of feathers could be greater in colder regions, while colour variation may also play a role in adapting to local environmental conditions since darker feathers are known to absorb more heat than lighter ones. We studied feather properties (plumulaceous part of the feather, density of barbs and barbules) of tawny owl, *Strix aluco* , across nine populations covering a large part of the species' European range (9–52 individuals per population) as well as their plumage colour, scored as dark (brown) versus light (grey) morphs. We compared these traits' phenotypic divergence (P_ST_) with the divergence expected based on genetic drift (F_ST_) inferred using eight microsatellites. The F_ST_ was low (0.022; 95% CI 0.005–0.039), and most feather structures' phenotypic divergence (P_ST_) exceeded the F_ST_. However, phenotypic divergence in plumage colour was low and not significant, implying a limited role of natural selection in shaping variation in plumage colouration at large spatial scales. Between‐population differentiation in feather properties was more pronounced in ventral feathers than dorsal feathers. In colder populations, the plumulaceous part of the dorsal feathers, but not the ventral feathers, was larger (implying greater insulation). Although proper evaluation hinges on understanding how insulative properties confer a fitness advantage in a given environment, our findings imply that properties of avian feathers may reflect local adaptation, possibly related to climate.

## Graphical abstract

Populations of tawny owls may exhibit local adaptations in feather properties, with variations in insulation and colouration linked to environmental factors like temperature. Our study across nine European populations showed significant phenotypic divergence in feather structures, but minimal divergence in plumage colour, suggesting limited natural selection's role in shaping colouration at larger spatial scales. The findings indicate that feather properties, particularly in colder regions, may reflect local adaptation, possibly related to climate, though further research is needed to assess their functional role in fitness.

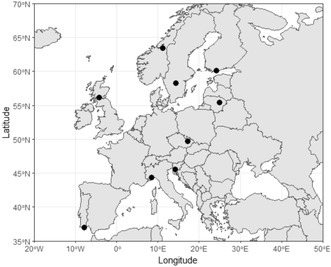

## 1 Introduction

Populations are expected to show divergence as an adaptation to the specific climatic and environmental conditions they experience, such as, for example, weather and seasonality, as well as vegetation and resource availability (Parmesan 2006; Gilg et al. 2012; Shaw and Etterson 2012; Thorpe et al. 2015). Traits favorable under specific climatic and environmental conditions will be selected, leading to differentiation across populations (Fisher 1958; Bulmer 1985; Hill and Mackay 2004). Differentiation between populations can cause genetic drift by stochastically changing the frequency of an existing gene variant (Lande 1976; Masel 2011; Lynch et al. 2016). In isolated populations, genetic drift can reduce genetic diversity within a population but increase between‐population genetic differentiation. If populations are not isolated, gene flow between them will counteract genetic drift's effect on between‐population differentiation but may also disrupt local adaptation by homogenizing genetic variation across populations (Spieth 1974; Slatkin 1985; Lenormand 2002).

The classic approach to study population differentiation in quantitative traits is to rear organisms of various populations in a common environment, as any between‐population differences in these traits then do not reflect environmental differences between these populations but instead presumably have a genetic basis. Using in situ measurements to quantify P_ST_ (Phenotypic divergence) of focal traits is a potentially interesting alternative for species that are challenging to rear in a common environment, such as birds. Indeed, it can be used to identify phenotypically differentiated traits that could be worthwhile candidates for further in‐depth investigation into local adaptation (Brommer 2011). When between‐population phenotypic differentiation (P_ST_) in a trait exceeds the expectation of neutral divergence (F_ST_, Fixation index, measure of population differentiation due to genetic structure), it can be considered as a signature of local adaptation, although—of course—it cannot be excluded that the observed phenotypic differentiation is caused exclusively by environmental differences rather than genetic differences. Local adaptation means differences between populations are due to genotypes, by definition. However, as we measure phenotypes in their natural environment, all observed differences in phenotype (within and across populations) can be due to plasticity; that is, the direct effect of the environment on the traits measured.

When considering birds, feathers are an attractive trait for studying local adaptations because they have many different physiological and behavioural functions (Dyck 1985; Terrill and Shultz 2023). Feather functions include flight, sexual selection, camouflage, and insulation. Feathers can be widely different in morphology and structure depending on their function. A feather is composed of a rachis to which serial paired branches, so‐called barbs, are attached (Prum 1999). The barbs possess further branches, and the barbules of adjacent barbs are connected by hooks (Prum 1999). Body feathers are broadly defined as the stiff outer layer of the integument that protects the skin in birds. These feathers are most numerous in terms of their numbers and mass (Wetmore 1936; Davenport et al. 2009) and are critical for thermal insulation and waterproofing (Davenport et al. 2009; Williams et al. 2015). The number of barbs and barbules determines the insulation capacity of a plumage (Stettenheim 2000). Generally, bird species living in cold environments have longer feathers with longer plumulaceous parts of the feather (Barve et al. 2021) and lower barb and barbule densities to trap more air (Pap et al. 2017). Thus, plumage insulation capacity across species appears to covary with climatic conditions.

In addition to insulation, the colour of the feather affects heat absorption (Rogalla et al. 2022). Darker feather surfaces absorb more heat than light‐coloured surfaces under exposure to the sun due to the properties of melanin pigments present in the feathers. Thus, the insulation capacity of darker feathers could be less than that of lighter feathers since they absorb more heat. Darker plumage birds would be selected to produce less costly, that is, shorter plumulaceous feathers since they already benefit from heat absorption. Insulation and heat absorption are essential, especially for species living in climatic areas with harsh winters, like the tawny owl. Tawny owls are widely spread across Europe and occupy different habitats and climate zones. Tawny owl plumage colouration is highly heritable (h2 = 72%) and can be characterised in a grey (light) morph and a brown (dark) morph (Brommer et al. 2005). According to Gloger's rule, birds and mammals are expected to be darker in humid and warm environments compared to colder and drier areas (Delhey 2017). Tawny owls at least partly adhere to this biogeographical pattern across Europe, as the proportion of brown tawny owl morphs in a population is higher when winters are warmer but declines when winters are wetter and summers are warmer (Koskenpato et al. 2023). Tawny owl plumage colouration is linked to overwinter survival in a Finnish population, where brown tawny owls' survival is lower than grey tawny owls' survival when the snow depth is high (Karell et al. 2011). As winter got warmer with less heavy winters with lots of snow, the frequency of the brown morph increased in this population (Karell et al. 2011). Moreover, considering three colour morphs (light, dark and an intermediate morph), the intermediate‐coloured tawny owl morph increased in a Lithuanian population over time, implying a change in the survival propensity of the different morphs (Grašytė et al. 2017). In a Finnish study population of tawny owls, dorsal feathers exhibited higher insulation capacity in grey owls compared to brown owls (Koskenpato et al. 2016). This difference in insulation may contribute to grey tawny owls surviving better in cold winters (Karell et al. 2011).

Within species, still little is known about how feathers could be adapted to certain types of environments. The proportion of plumulaceous on dorsal feathers increased along an elevational climate gradient in house sparrows, presumably linked to climatic conditions (Barve et al. 2021). Broggi et al. (2011) showed that great tits from a northern latitude population had denser plumage but consisted of shorter feathers with a smaller proportion containing plumulaceous barbs compared with conspecifics from the southern population. A reciprocal transplant experiment between these populations shows a partly genetic basis for these differences, suggesting local adaptation (Broggi et al. 2011). Hence, there is some evidence that feather and plumage characteristics result from adaptation to local environmental conditions, and a better understanding of the adaptive capacity of these characteristics can also be essential in the context of climate warming, which results in changing selective pressures.

Here, the aim is to understand if the insulation properties of tawny owl body feathers are morph and/or location‐dependent, using feathers collected in nine different European populations. We predict that tawny owls living in colder environments have longer feathers but lower barb and barbule density, as well as a longer plumulaceous portion in the feather, given that birds living in colder environments are expected to have such feather characteristics (Pap et al. 2017; Barve et al. 2021). Furthermore, darker feather surfaces absorb more heat than light‐coloured surfaces under exposure to the sun (Rogalla et al. 2022). And since dorsal feathers have a lower insulation capacity in brown owls than in grey owls in Finland (Koskenpato et al. 2016), we expect that, across all our study populations, brown tawny owls would have a higher density of barbs and barbules and a shorter plumulaceous portion of their feathers (i.e., lower insulation capacity) compared to grey tawny owls. We compute the between‐population differentiation in phenotypes (P_ST_) of measured feather properties and compare it to the differentiation expected based on genetic drift alone (F_ST_) to evaluate which, if any, of tawny owl feather properties show evidence of local adaptation. However, the environmental correlation component is missing, which limits our ability to build strong arguments regarding local adaptation.

## 2 Material and Methods

### 2.1 Feathers' Structure Measurements

**FIGURE 1:**
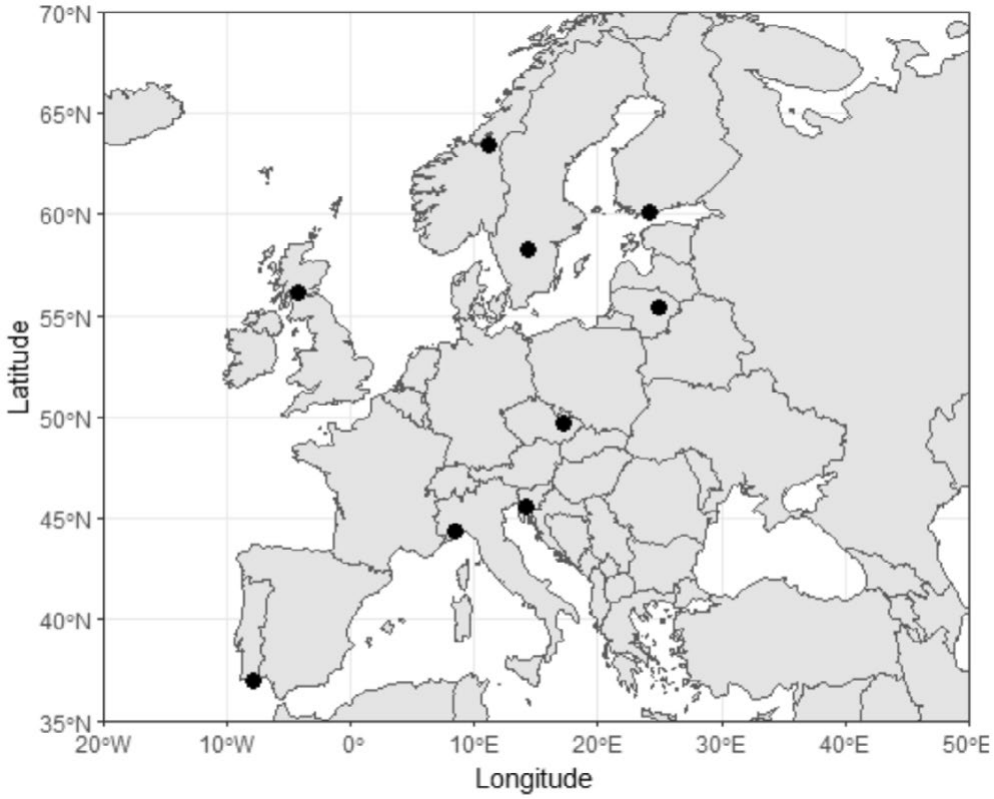
Map of the nine different populations distributed across Europe, the populations' positions are represented with black dots.

Feathers from nine different populations of tawny owls across Europe were collected (Figure 1) between 2015 and 2024. The populations are from Finland, Sweden, Norway, Scotland, Italy, Portugal, Slovenia, Czech Republic, and Lithuania. Most of the populations data come from population monitoring during fieldwork, except for the Portugal population, which came from a wildlife hospital (Wildlife Rehabilitation and Research Center—RIAS (Olhão)). A central point for each population was determined, such as: Finland (central point Siuntio: 60°15′N, 24°15′ E), Sweden (central point Skaraborg: 58°31′N, 14°31′ E), Norway (central point Levanger: 63°43′ N, 11°21′ E), Lithuania (central point Kavarskas: 55°43′N, 24°92′ E), Czech Republic (central point Olomouc: 49°69′ N, 17°15′ E), Italy (central point Cairo Montenotte: 44°37′ E, 8°36′), Portugal (central point Faro: 36°98′N, −7°92′ E), Scotland (central point Aberfoyle: 56°17′N, −4°36′ E) and Slovenia (central point Mt. Krim: 45°58′N, 14°25′ E).

**FIGURE 2:**
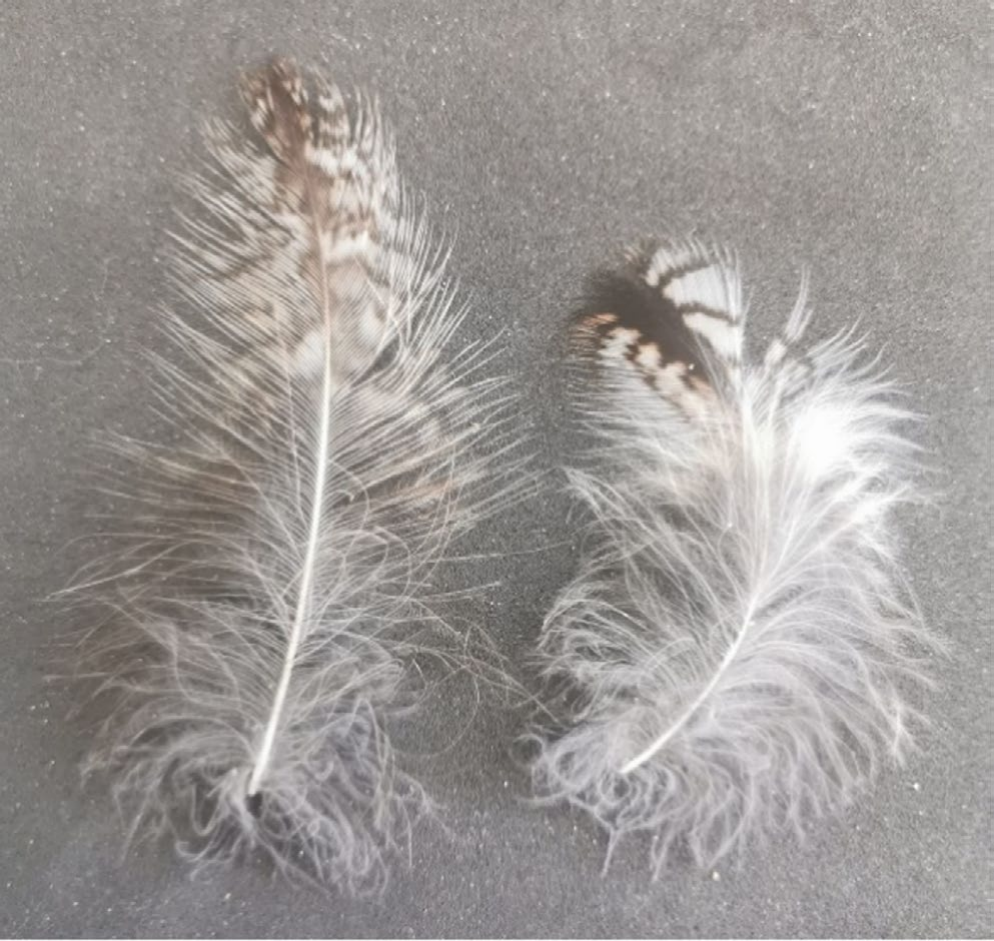
Picture of a dorsal feather (left) and a ventral feather (right).

**FIGURE 3:**
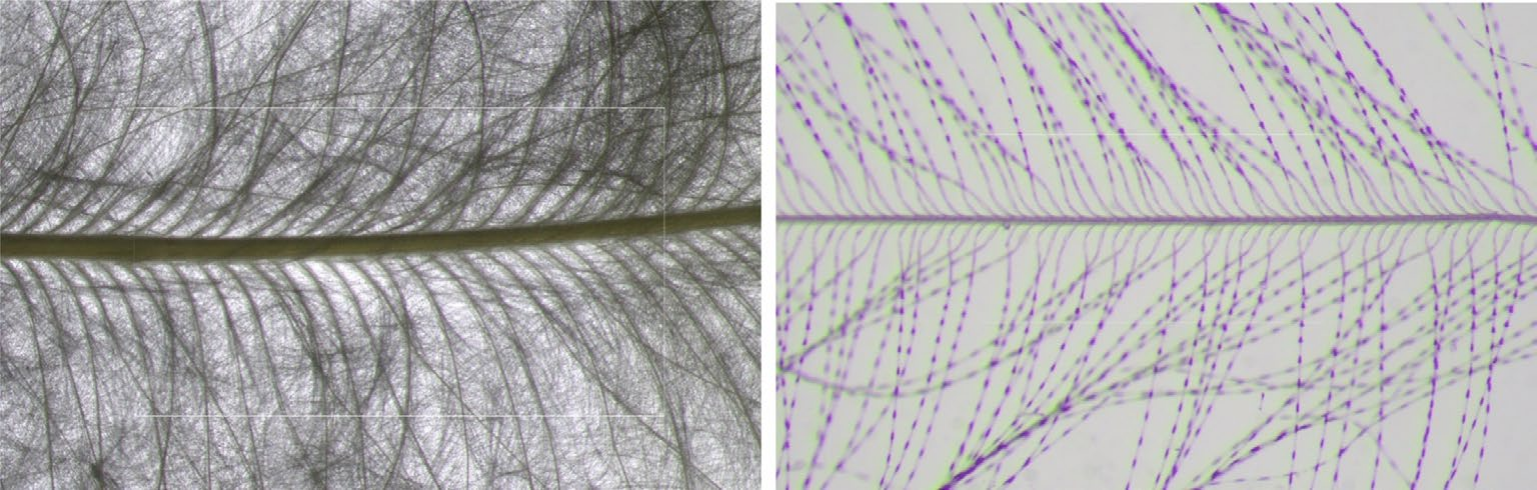
Microscope pictures of a feather (left, × 10 magnification) and of a barb (right, × 63 magnification).

Four feathers per individual were requested: two feathers from the dorsal side (Figure 2) and two feathers from the ventral side (Figure 2). In some cases, only one feather for the dorsal side or ventral side was provided, or only one feather category was provided; see results (Table 1). Each feather was measured by hand with a ruler, and its plumulaceous part was without the rachis. Microscope pictures were taken from the feathers and from one barb from each feather (Figure 3). The microscope used was a Leica MZ10F Stereo. We used the magnification × 10 for the feather pictures and × 63 for the barb pictures. Each time, the picture was taken from the middle part of the feather or the barb. A square of 1 cm for barbs and 1 mm for barbules was drawn on each picture at the stem level with ImageJ image analysis software (Schindelin et al. 2015) using a scale defined according to the microscope; the pixel distance was converted into centimetres (1 cm = 950 pixels, 1 mm = 765 pixels). One observer counted the number of barbs and barbules of feathers on the microscope pictures within the drawn square from five populations out of nine, and another observer counted on the microscope pictures from the remaining four populations out of nine. Two observers counted barbs and barbules for a subset of 39 feathers from random populations. The agreement between the counts made by these two observers was very high (barbs: cor = 0.95, *p* value < 0.001, *N* = 19; barbules: cor = 0.99, *p* value < 0.001, *N* = 20).

**TABLE 1:** Sample size of feathers (dorsal and ventral), the number of individuals per population, the number of individuals successfully amplified per population, the proportion of grey individuals per population, and the years of collection.

	Dorsal feathers	Ventral feathers	Individuals	Individuals successfully amplified	Proportion of grey individuals	Year of collection
Czech Republic	189	189	52	25	0.39	2021
Finland	51	51	24	16	0.55	2021, 2020
Italy	52	50	18	12	0.62	2024, 2023, 2022, 2021
Lithuania	136	136	37	3	0.46	2021
Norway	120	129	34	18	0.51	2022, 2018
Portugal	14	16	9	10	0.47	2024, 2023, 2022, 2021
Scotland	43	10	21	8	0.13	2023, 2022
Slovenia	49	46	27	7	0.46	2023, 2021, 2020, 2019, 2018, 2017, 2016, 2015
Sweden	161	162	43	0	0.33	2022, 2021, 2019, 2020

### 2.2 Colour Scoring of the Owls

The scale involves scoring the owl's colouration based on facial disc, back, belly, and overall appearance. A final score between 4 (very light grey) and 14 (very reddish brown) was determined. Italy's population was scored using the same colour‐scoring method as Finland. Tawny owls' morphs from Scotland and Portugal were determined using the same colour scoring method as those of the Finnish population, using pictures of each individual. The other populations (Sweden, Norway, Slovenia, the Republic of Czech and Lithuania) were originally colour‐scored using a different system with several morphs identified. However, individuals were then scored using the same method as in the Finnish population. When the morph “rusty” was used, we considered those individuals brown.

### 2.3 Climate Variables

For each central point between 2010 and 2020, we extracted the mean winter temperatures (in degree celsius, monthly mean surface air temperature for December, January and February) and the mean winter precipitations (in mm, for December, January and February) from ClimateCharts (Zepner et al. 2021) using CHCN CAMS and GPCC (Fan and van den Dool 2008).

### 2.4 Sampling, DNA Extraction

DNA utilised in this study stemmed from multiple tissues, primarily due to constraints in performing invasive sampling (collection of blood, muscle tissue, etc.) imposed on birds across the countries from which tawny owl populations were originally. Thus, DNA from individuals originally from Slovenia, Scotland, Norway, Italy, and the Czech Republic was extracted from buccal swabs (IsoHelix) that sampled epithelial cells in tawny owls' mouths; DNA from individuals originally from Finland was extracted from fresh blood maintained in a non‐coagulation solvent; DNA from Lithuania was extracted via dry blood droops preserved in filter paper. The only exception was Portugal, where DNA was obtained from muscle tissue. This is because our collaborator in the country was a Wildlife Rehabilitation and Research Centre that granted access to their deceased owls. A standardised DNA protocol was established across samples, which consisted of a customised version of the salt extraction protocol (Aljanabi and Martinez 1997) to increase DNA yield. All samples were genotyped with eight microsatellite markers using a multiplex PCR approach (Thode et al. 2002; Saladin et al. 2007). PCR was carried out in three 12 μL reactions using a QIAGEN Multiplex PCR Kit (Qiagen Inc. Valencia, CA, USA) with an annealing temperature of 55°C and a primer concentration of 0.2 μM. To improve the microsatellite peak profiles, a GTTT‐tail was added to the 5′ end of each reverse primer (Brownstein et al. 1996), as seen in Table S1. Amplifications were performed on Applied Biosystems 2720 thermal cyclers, and the size of the fragments was determined by capillary electrophoresis on an ABI Prism 3130xl genetic analysis instrument. The peak profiles of the pooled samples could then be separated during scoring and visual inspection using GeneMarker version 2.4.0 (SoftGenetics). Laboratory work for microsatellite genotyping was carried out by the Center of Evolutionary Applications (University of Turku, Finland).

### 2.5 Ethics Statement

For our Scottish population, the feather sampling and buccal swabs were conducted under licence (BTO Special Methods) 12145, 12146. The sampling protocol, including plucking two feathers from the dorsal and ventral sides of a tawny owl during ringing, as well as buccal swabs, is not considered animal experiments in the EU. Blood sampling in Finland was performed under licence by the Regional State Administrative Agency (ELLA) ESAVI/15854/2022. Blood sampling in Lithuania was conducted under that permission number (26)‐SR‐156.

### 2.6 Statistical Analyses

All feather traits were analysed using mixed models using R software version 4.1.2 (2021‐11‐01) (http://www.R‐project.org/). The model included the year of sampling as factorial fixed effects, and for the length of the plumulaceous part of the feather, we also included the total feather length. Initial models were constructed, including all levels of repeated measures as random effects. For the number of barbs and barbules, we included population and ID of the sampled individual as random effects. For the length of the plumulaceous feather, we included the population, ID of the sampled individual, and ID of the measurement. Thus, these models partitioned the variance (conditional on the fixed effects) into between‐population, between‐individual, and (for plumulaceous feathers) between repeated measures on the same individual. To compute the differentiation between populations (PST), we used the mean values for the number of barbs and barbules and the mean length of the plumulaceous part of the feather as response variables for each individual overall measure. Fixed effects in the model were as described above, except that the mean total feather length was used as a fixed effect in the analysis of the plumulaceous part of the feathers. The random effect included was population, and P_ST_ was computed as follows:

(1) $$P_{\mathrm{ST}}=\frac{\sigma^{2}\left(\mathrm{pop}\right)}{\sigma^{2}\left(\mathrm{pop}\right)+\sigma^{2}\left(\mathrm{res}\right)}$$

where *σ* ^2^(pop) and *σ* ^2^(res) are the between population and residual REML variances respectively as inferred by the above described mixed model.

Mean values for each individual were calculated for the number of barbs and barbules. Models were implemented in the package sommer assuming a Gaussian distribution (Covarrubias‐Pazaran 2016) in R, except for the differentiation of plumage colour morphs (brown coded as 1; grey as 0) across populations, which was analysed in a mixed model with binomial error distribution and logit link implemented in AsReml (VSN International). The proportion of variance in colour morphs across the population was computed on the logit scale following Nakagawa and Schielzeth (2010). Uncertainty (standard error) of the ratio of variances—such as P_ST_ (Equation 1) or the proportion of variance due to between‐individual variance (i.e., repeatability)—was inferred using the delta method as implemented in the statistical packages used.

For the genetics, population level statistics were calculated in Arlequin v3.5, based on a pairwise distance matrix considering only markers with < 20% missing data and with 10,000 permutations (Excoffier and Lischer 2010).

## 3 Results

Table 1 indicates the number of feathers by type (ventral or dorsal), the number of individuals, and the proportion of grey individuals among populations.

### 3.1 Genotyping and Estimates of Population Differentiation

A total of 99 individuals were successfully amplified by our microsatellite panel, with substantial variation in terms of population‐specific success (see Table 1). Population differentiation estimates were only calculated as an average (global) F_ST_ of 0.022 (SD ± 0.026), given that the purpose was simply to obtain a global estimate of F_ST_ and build expectations on how much variation in phenotypic traits can be attributed to populations evolving in isolation without natural selection (drift).

### 3.2 Plumulaceous Length

**FIGURE 4:**
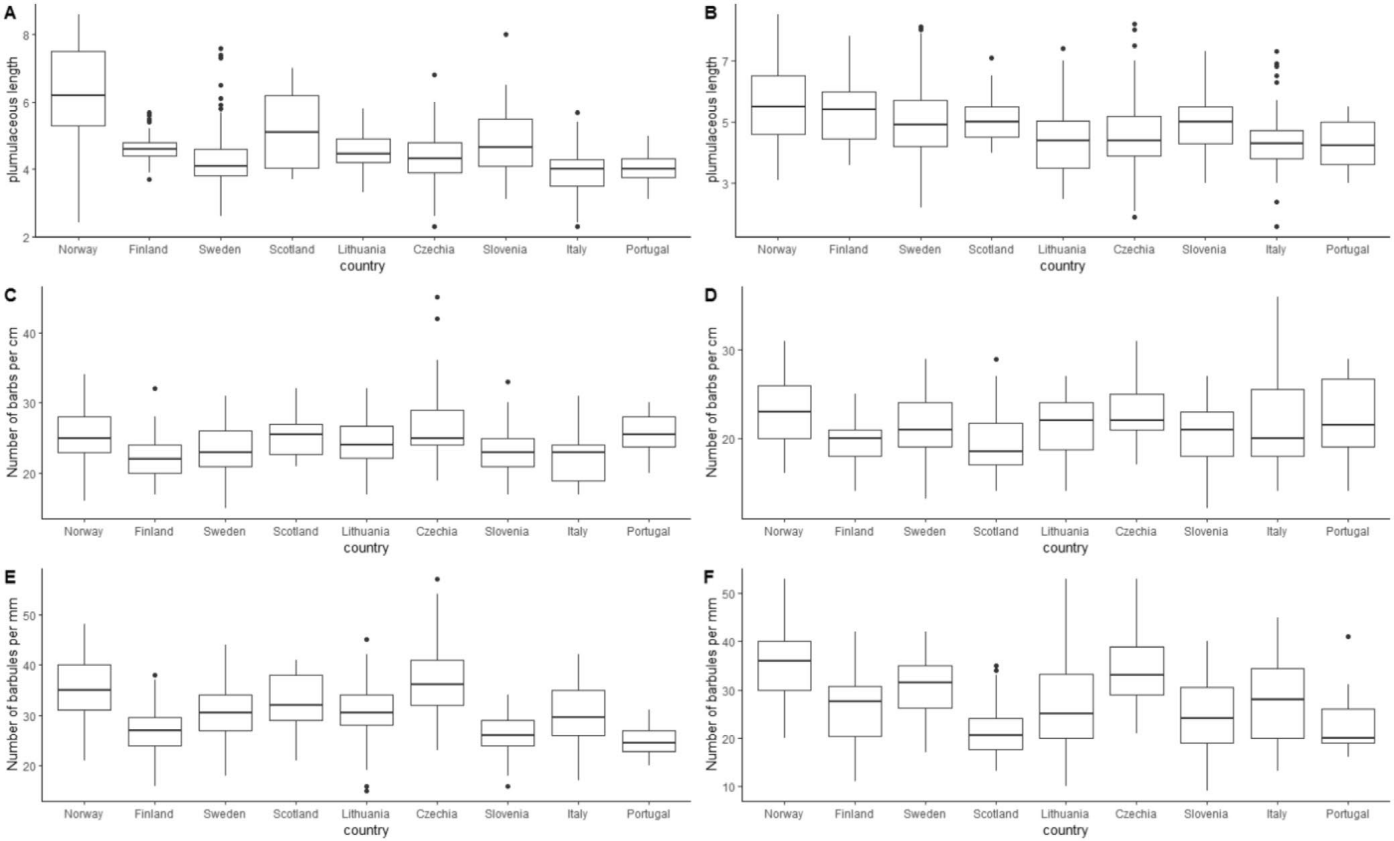
Boxplots of raw data representing the differences between all the countries (ordered by latitude from northern to southern point) for: Plumulaceous length for ventral (A) and dorsal (B), number of barbs per cm for ventral (C) and back (D), and number of barbules per mm for ventral (E) and dorsal (F) barbs on ventral feather and plumage colouration.

The plumulaceous length of feathers varied significantly across populations in both ventral and dorsal feathers, with population differences explaining 39% of the total variance in ventral feathers (*χ* ^2^ = 53.25, *p* < 0.001) and 16% of the total variance in dorsal feathers (*χ* ^2^ = 18.54, *p* < 0.001) (Table 2, Figure 4). The total feather length correlated significantly with the plumulaceous length for both the dorsal side and ventral side (Table 2). Grey tawny owls had a shorter plumulaceous length of the feathers on their dorsal side (Table 2) but not their ventral side. There were some differences in the plumulaceous length of feathers between years, with the year differences explaining 8%–9% of the total variance for both ventral and dorsal plumulaceous length (Table 2).

**TABLE 2:** Linear mixed model analysis of the length of plumulaceous feather (ventral feathers, *n* = 246; dorsal feathers, *n* = 263).

Plumu ventral average	Var	SE	Prop V	SE	LRT	
					Chi‐squared	Pr
Random effects						
Residuals	0.12	0.01	0.53	0.12		
**Population**	**0.09**	**0.05**	**0.39**	**0.14**	**53.25**	**< 0.001**
**Year**	**0.02**	**0.02**	**0.08**	**0.07**	**149.27**	**< 0.001**

Fixed effects	Estimate	SE	Chi‐square	df	Pr
**Feather length scaled**	**0.84**	**0.03**	**592.26**	**1**	**< 0.001**
Morph G	0.02	0.05	0.15	1	0.70

Plumu dorsal average	Var	SE	Prop V	SE	LRT	
					Chi‐square	Pr
Random effects						
Residuals	0.28	0.03	0.75	0.10		
**Population**	**0.06**	**0.04**	**0.16**	**0.09**	**18.54**	**< 0.001**
**Year**	**0.03**	**0.03**	**0.09**	**0.08**	**159.53**	**< 0.001**

Fixed effects	Estimate	SE	Chi‐square	df	Pr
**Feather length scaled**	**1.04**	**0.05**	**470.51**	**1**	**< 0.001**
**Morph G**	**−0.15**	**0.07**	**4.55**	**1**	**< 0.05**

*Note:* The population and the year of collection are considered as random factors with their variance (Var) with its standard error (SE) as well as the proportion of variance (prop V) and its SE denoted. A Likelihood Ratio Test (LRT) was used to test for statistical significance of each random effect and it reported as chi‐square tested and its associated *p* value with one degree of freedom. As fixed effects, the morph of the owl (grey vs. brown) and the total feather length (scaled to zero mean and unit variance) were included and the estimate with its SE as well as the Wald chi‐square test statistics are reported for these fixed effects. Significant terms are indicated in bold font.

### 3.3 Number of Barbs

The number of barbs on ventral feathers varies significantly across populations (Table 3, Figure 4) but not on dorsal feathers (Table 3, Figure 4). There are no significant differences in the number of barbs between the two colour morphs (Table 3).

**TABLE 3:** Linear mixed model analysis of the number of barbs (ventral feathers, *n* = 246; dorsal feathers, *n* = 261).

Barbs ventral average	Var	SE	Prop V	SE	LRT	
					Chi‐square	Pr
Random effects						
Residuals	10.32	0.96	0.81	0.1		
**Population**	**1.68**	**1.24**	**0.13**	**0.09**	**15.8**	**< 0.001**
Year	0.76	0.83	0.06	0.08	1.16	0.28

Fixed effects	Estimate	SE	Chi‐square	df	Pr
Morph G	−0.02	0.42	0.003	1	0.96
Researcher SK	0.59	0.99	0.36	1	0.55

Barbs dorsal average	Var	SE	Prop V	SE	LRT	
					Chi‐square	Pr
Random effects						
Residuals	10.15	0.91	0.94	0.05		
Population	0.33	0.41	0.03	0.04	1.97	0.16
Year	0.27	0.51	0.02	0.05	0.76	0.38

Fixed effects	Estimate	SE	Chi‐square	df	Pr
Morph G	−0.26	0.41	0.41	1	0.52
**Researcher SK**	**2.33**	**0.62**	**14.11**	**1**	**< 0.001**

*Note:* The population and the year of collection are considered as random factors with their variance (Var) with its standard error (SE) as well as the proportion of variance (prop V) and its SE denoted. A Likelihood Ratio Test (LRT) was used to test for statistical significance of each random effect and it reported as chi‐square tested and its associated *p* value with one degree of freedom. As fixed effects, the morph of the owl (grey vs. brown) and the identity of the measurer were included and the estimate with its SE as well as the Wald chi‐square test statistics are reported for these fixed effects. Significant terms are indicated in bold font.

### 3.4 Number of Barbules

The mean number of barbules for both dorsal and ventral feathers is significantly correlated with populations (Table 4, Figure 4). There are no significant differences in the number of barbules between the two colour morphs (Table 4).

**TABLE 4:** Linear mixed model analysis of the number of barbules (ventral feathers, *n* = 246; dorsal feathers, *n* = 260).

Barbules ventral average	Var	SE	Prop V	SE	LRT	
					Chi‐square	Pr
Random effects						
Residuals	23.62	2.2	0.77	0.11		
**Population**	**6.65**	**4.24**	**0.22**	**0.11**	**39.87**	**< 0.001**
Year	0.35	1.42	0.01	0.05	0.12	0.73

Fixed effects	Estimate	SE	Chi‐square	df	Pr
Morph G	−0.6	0.64	0.89	1	0.35
**Researcher SK**	**6.28**	**1.55**	**16.49**	**1**	**< 0.001**

Barbules dorsal average	Var	SE	Prop V	SE	LRT	
					Chi‐square	Pr
Random effects						
Residuals	39.42	3.56	0.85	0.09		
**Population**	**6.13**	**4.28**	**0.13**	**0.08**	**19.25**	**< 0.001**
Year	0.83	2.21	0.02	0.05	0.37	0.54

Fixed effects	Estimate	SE	Chi‐square	df	Pr
Morph G	0.11	0.81	0.02	1	0.9
**Researcher SK**	**10.1**	**1.71**	**34.89**	**1**	**< 0.001**

*Note:* The population and the year of collection are considered as random factors with their variance (Var) with its standard error (SE) as well as the proportion of variance (prop V) and its SE denoted. A Likelihood Ratio Test (LRT) was used to test for statistical significance of each random effect and it reported as chi‐square tested and its associated *p* value with one degree of freedom. As fixed effects, the morph of the owl (grey vs. brown) and the identity of the measurer were included and the estimate with its SE as well as the Wald chi‐squre test statistics are reported for these fixed effects. Significant terms are indicated in bold font.

### 3.5 Proportion of Variance Between Populations P_ST_

**FIGURE 5:**
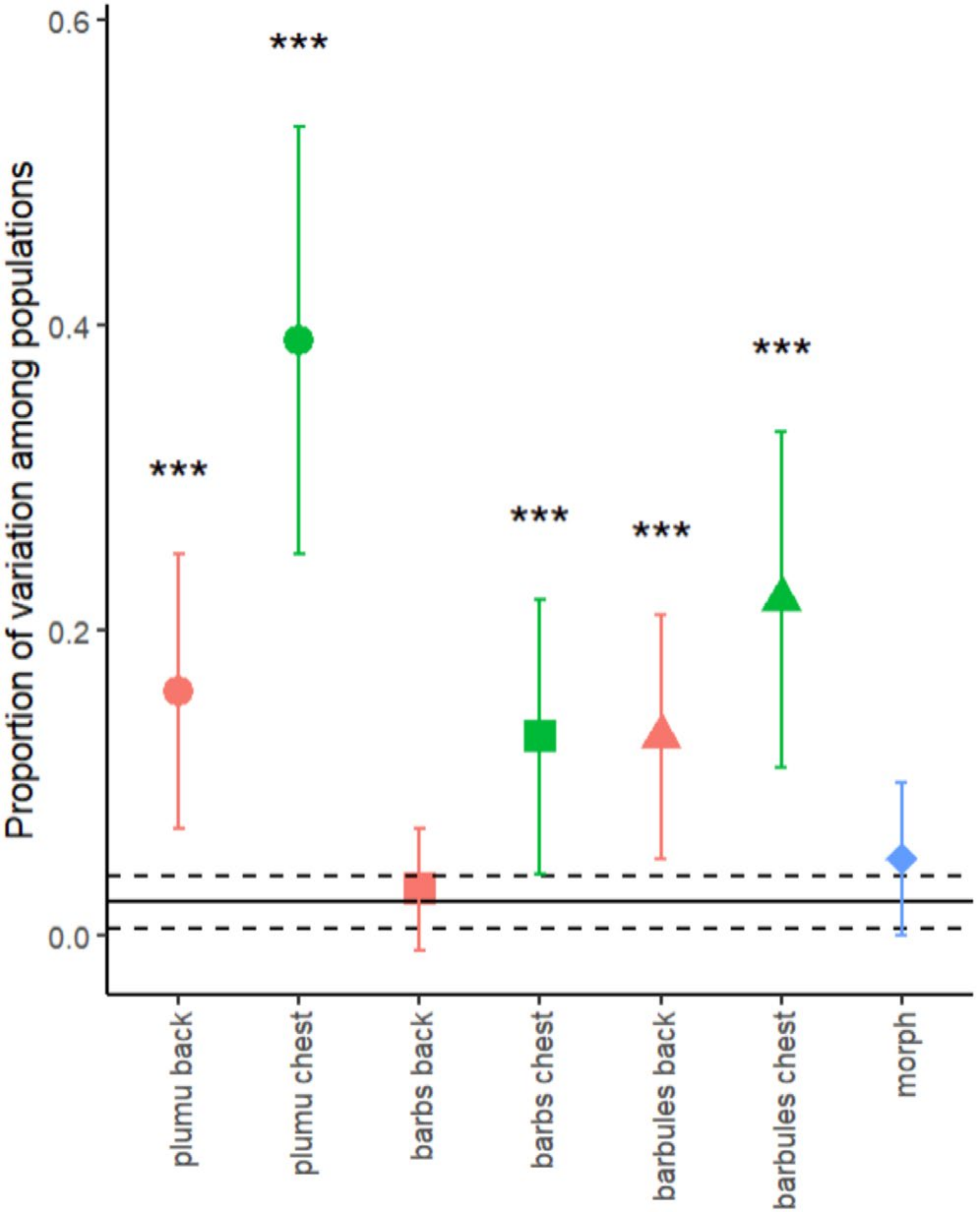
The different proportion of variance between populations (P_ST_) and their standard errors for the plumulaceous length (filled circles), the number of barbs (filled squares) and barbules (filled triangles). The green colour indicates the ventral feathers, the orange colour the dorsal feathers and the blue colour the morph of individuals. Stars are indicating significance in the LRT test with “***” meaning *p* < 0.001. The F_ST_ is represented with a horizontal line and the dotted lines represents the 95% confidence interval of the F_ST_.

All feather characteristics except the density of barbs on the dorsal side showed significant variation across populations (Tables 2, 3, 4, Figure 5). Overall, the proportion of variance explained by population differences (P_ST_) was highest for the length of the plumulaceous part of ventral feathers (P_ST_ of 42%) but was more modest for the other feather characteristics. The average morph proportion was 60% brown (inverse logit of the mixed model's intercept, Table 5) across these populations. The binomial mixed model inferred the variance of morphs across populations. Still, this variance also had substantial uncertainty (large SE) and was not significant (based on the Z ratio test of the estimate, Table 5, Figure 5). The proportion of variance across populations (P_ST_) in morph on the logit scale was 5% (Table 5, Figure 5), indicating a very low level of differentiation that was not significantly different from zero. The point estimate of P_ST_ exceeded the upper 95% confidence interval of the F_ST_ estimate for all feather properties except for the traits that showed no significant between‐population differentiation (density of barbs on ventral feather and the plumage colouration).

**TABLE 5:** Mixed model with binomial errors on the probability for individuals to have brown plumage colour (scored as 1) vs. grey plumage colour (scored as 0).

MORPH	Var	SE	Prop V	SE	Z	*p*
Random effects						
Population	0.18	0.19	0.05	0.05	0.98	0.59
Year	0.03	0.13				

Fixed effects	Estimate	SE
Intercept	0.41	0.22

*Note:* Variance is partitioned into an across populations and across years component. Reported for the random effects are the variance inferred (Var) with its standard error (SE), the proportion of phenotypic variance (Prop V) on the logit scale with its standard error (SE) as well as the Z and *p* value for the variance estimate differing from zero. The model included only the intercept and reported here is its estimate with associated standard error on the logit scale.

### 3.6 Correlation of P_ST_ With Temperature and Precipitation

**FIGURE 6:**
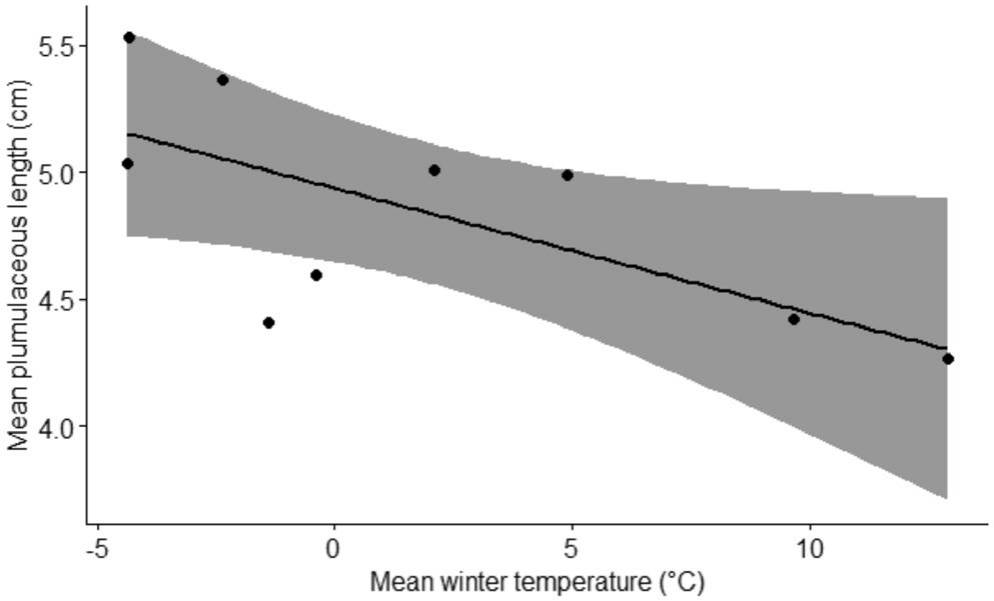
Correlation regression between the mean winter temperature in degree celsius and the mean plumulaceous length of dorsal feathers in cm.

The plumulaceous length of dorsal feathers was correlated with the mean winter temperature. The mean plumulaceous length of dorsal feathers increases as the mean winter temperature decreases (Figure 6, Table 6). None of the other feather traits were correlated with the mean winter temperature (Table 6). None of the feather traits were correlated with mean precipitation values (Table 6).

**TABLE 6:** Pearson's product–moment correlation results for the correlations between mean winter temperature and feathers structures variables as well as the correlations between mean winter precipitation and feathers structures variables.

	Mean winter temperature		Mean winter precipitation	
	Cor	*p* value	Cor	*p* value
**Mean plumulaceous length dorsal**	**−0.68**	**0.04**	0.33	0.39
Mean plumulaceous length ventral	−0.51	0.16	0.38	0.31
Mean number of barbs dorsal	0.007	0.99	−0.57	0.11
Mean number of barbs ventral	0.02	0.97	0.05	0.89
Mean number of barbules dorsal	−0.53	0.14	−0.47	0.21
Mean number of barbules ventral	−0.53	0.14	0.004	0.99

*Note:* Significant results are reported in bold.

## 4 Discussion

Our findings show that tawny owl body feathers differ in several properties across European populations. We find that tawny owl populations differ in the size of the plumulaceous part of the feather and in microscopic feather properties such as the densities of barbs (except for dorsal feathers) and density of barbules. The differentiation based on microsatellites between the European tawny owl populations is low (F_ST_ = 0.022; 95% CI 0.005–0.039), implying substantial gene flow between populations consistent with the generally low differentiation observed among bird populations (Barrowclough 1980). Thus, we expect a low differentiation in phenotypic traits based on neutral divergence processes alone. The differentiation in these feather properties across populations as measured by P_ST_ varies from 14% to 42%, thus clearly exceeding the expected neutral differentiation (F_ST_). On the other hand, plumage colouration differentiation between our populations is not different from what is expected with genetic drift (P_ST_ on the logit scale is 5%).

Our finding of population differentiation in most feather properties exceeding the neutral divergence expectation suggests that these properties may have been shaped by selection, indicating potential local adaptation. All these feather properties affect the plumage's insulation capacity, suggesting that populations may be adapted to their local climatic conditions. However, we find that only the plumulaceous part of the dorsal feathers decreases in populations with higher mean winter temperatures, suggesting that aspects other than winter climate are important factors driving inter‐population differentiation among feather properties. Nevertheless, a weakness of the P_ST_—F_ST_ comparison used here is that much (potentially all) of the observed differences between populations can be directly caused by the difference in the environmental conditions between the populations rather than produced via natural selection in adapting to that environmental condition. Furthermore, we lack information on the genetic basis of these feather traits, hampering an assessment of their potential to adapt.

Between‐population differentiation in feather properties was more pronounced in ventral feathers than in dorsal feathers. The plumulaceous part of the dorsal side, but not the ventral side, was larger (implying greater insulation) in colder populations, where the Norwegian population especially stands out with a longer plumulaceous part of the feather compared to the other populations. However, only dorsal feathers showed variation following a north–south gradient, with the northern (colder) populations having a longer plumulaceous part than the southern (warmer) populations. This finding is in accordance with birds living in colder environments having longer feathers with longer plumulaceous portions in the feather (Pap et al. 2017; Barve et al. 2021). The plumulaceous length seems to be an essential feather feature for birds to have better insulation in harsh winters. One possible explanation for why only the plumulaceous length of dorsal feathers is correlated with winter temperatures may be that, as birds' lungs are closer to their dorsal side than their ventral side (Farner and King 2013), better insulation in the dorsal feathers could be essential to protect vital organs such as lungs from cold temperature, especially in harsh winters when breathing cold air. In addition, birds' ventral side and abdominal cavities carry fat storage cells that would help with front insulation (Blem 1976). Although proper evaluation hinges on understanding how insulative properties confer a fitness advantage in a given environment as well as on the demonstration of their genetic basis, our findings imply that properties of avian feathers may reflect local adaptation, possibly related to climate.

Birds living in colder environments have low barb and barbule density to trap more air (Pap et al. 2017; Barve et al. 2021). Our results show that populations only differ in the density of barbs in the ventral feathers. Finland, Slovenia, and Sweden seem to have a lower density of barbs in ventral feathers than other populations. Still, overall, the density of barbs was not correlated to climate across tawny owl populations. Interestingly, we find quite high between‐population differentiation in the density of barbules (23% and 13% for ventral and dorsal body feathers, respectively). Although this differentiation is not correlated with winter climatic conditions, it indicates how population‐specific factors are essential for the tawny owl's microscopic features of body feathers.

Darker feather surfaces absorb more heat than light‐coloured surfaces under exposure to the sun (Rogalla et al. 2022). In Finland, dorsal feathers have a lower insulation capacity in brown tawny owls than in grey tawny owls (Koskenpato et al. 2016). Based on these two findings, we expected brown tawny owls' feathers to provide lower insulation than those of grey tawny owls. Yet, our results show that tawny owl morphs only differ in their insulation capacity for the plumulaceous length in the dorsal feathers. Indeed, grey tawny owls seem to have shorter plumulaceous parts of the dorsal feathers than the brown tawny owls. Our finding based on samples of several European populations is thus contrary to the previous study made in Finland (Koskenpato et al. 2016). This difference in results between the latter study and ours could be due to the multiple observers in our study, the different statistical approach, the different sample sizes, and the different years of collection. We did not find significant differentiation across populations in tawny owl plumage colouration. The colouration does not explicitly follow Gloger's rule in tawny owls, suggesting a more complex system shaped by many factors (Koskenpato et al. 2023). Indeed, many confounding factors such as diet, habitat differences, or ontogenetic variations could influence feather traits morphology in each population. Nevertheless, as we considered plumage colouration here as a binomial phenotype, our sample size (number of individuals per population) was restrictive. On average, the ratio was about 50%:50%, meaning that the random binomial variance is maximal, thus making it challenging to detect differences between the populations. More individuals per population are needed to infer the proportion of light vs. dark morphs reliably.

Although presumably shaped by selection, feathers appear understudied in evolutionary studies. Our approach appears promising in identifying properties of feathers that could be shaped by adaptation, although more work is needed in the future. A better understanding of the genetic basis of these traits and replication of the same studies on other species will be required if evolution is to be convergent for these traits (Ng and Li 2018). Moreover, it is important to consider the absence of direct ecological validation of the proposed adaptive advantages of feather traits. Studies including experimental data on heat retention or energy expenditure in different climates are needed to validate our hypothesis suggesting that plumulaceous length influences insulation capacity. However, our study shows that collaboration over large spatial scales is possible and opens the opportunity to study other trait differentiation or feather differentiation for multiple bird species. Museum samples, for instance, could offer good opportunities for large‐scale studies on feather differentiation within or in between populations/species.

## Author Contributions

**Charlotte Perrault:** data curation (equal), formal analysis (lead), funding acquisition (lead), investigation (lead), methodology (lead), software (lead), visualization (lead), writing – original draft (lead), writing – review and editing (lead). **Miguel Baltazar‐Soares:** data curation (equal), formal analysis (supporting), investigation (supporting), writing – review and editing (supporting). **Chiara Morosinotto:** investigation (equal), supervision (equal), validation (equal), writing – review and editing (equal). **Patrik Karell:** data curation (equal), investigation (equal), supervision (equal), validation (equal), writing – review and editing (equal). **Karel Poprach:** data curation (equal). **Ingar Øien:** data curation (equal). **Maria Casero:** data curation (equal). **Daniel Eriksson:** data curation (equal). **Peter Ericsson:** data curation (equal). **Gintarė Grašytė:** data curation (equal). **Katy Anderson:** data curation (equal). **Daniele Baroni:** data curation (equal). **Lars‐Ove Nilsson:** data curation (equal). **Saulius Rumbutis:** data curation (equal). **Jon E. Brommer:** conceptualization (lead), data curation (equal), formal analysis (equal), investigation (equal), project administration (equal), supervision (equal), validation (equal), writing – original draft (equal), writing – review and editing (equal).

## Conflicts of Interest

The authors declare no conflicts of interest.

## Supporting information


Table S1.

Figure S1.

## Data Availability

The data that support the findings of this study are openly available in Dryad at https://doi.org/10.5061/dryad.1vhhmgr4g.
