# Supplementary material for: Dressed for the Weather: Tawny Owl Feather Adaptations Across a Climatic Gradient
Source: Ecol Evol. 2025 Jun 24;15(6):e71441. doi: 10.1002/ece3.71441 (PMC12185932; doi:10.1002/ece3.71441)
Supplement: Supplementary file 1 — Table S1. [file ECE3-15-e71441-s002.docx]

|  |  |  |  |  | LRT | |
| --- | --- | --- | --- | --- | --- | --- |
| **PLUMU CHEST** | Var | SE | Prop V | SE | Chisq | Pr |
| random effects | | | | | | |
| residuals | 0,22 | 0,05 | 0,58 | 0,10 |  |  |
| **country** | **0,07** | **0,05** | **0,21** | **0,10** | **36,68** | **<0,001** |
| **id** | **0,04** | **0,01** | **0,11** | **0,03** | **20,83** | **<0,001** |
| **batch** | **0,04** | **0,04** | **0,11** | **0,03** | **68,59** | **<0,001** |
|  | Estimate | SE | df | Pr | Chisq | Pr |
| fixed effects | | | | | | |
| **feather length scaled** | **0,92** | **0,03** | **600,57** | **<0,001** |  | |
| morph G | 0,02 | 0,04 | 215,25 | 0,68 |  |  |
| **sex M** | **0,17** | **0,07** | **265,32** | **0,02** |  |  |
| sex U | -0,10 | 0,34 | 10,21 | 0,77 |  |  |
| Year 2016 | 0,31 | 0,39 | 396,47 | 0,43 |  |  |
| Year 2017 | 0,18 | 0,39 | 394,50 | 0,64 |  |  |
| **Year 2018** | **0,76** | **0,32** | **377,73** | **0,02** |  |  |
| Year 2019 | 0,02 | 0,33 | 354,71 | 0,95 |  |  |
| Year 2020 | 0,29 | 0,31 | 396,48 | 0,35 |  |  |
| Year 2021 | 0,27 | 0,30 | 395,28 | 0,36 |  |  |
| Year 2022 | 0,34 | 0,31 | 389,17 | 0,27 |  |  |
| Year 2023 | 0,48 | 0,30 | 403,82 | 0,10 |  |  |
| Year 2024 | 0,59 | 0,37 | 404,21 | 0,11 |  |  |

|  |  |  |  |  | LRT | |
| --- | --- | --- | --- | --- | --- | --- |
| **PLUMU BACK** | Var | SE | Prop V | SE | Chisq | Pr |
| random effects | | | | | | |
| residuals | 0,28 | 0,02 | 0,38 | 0,16 |  |  |
| country | 0,01 | 0,01 | 0,01 | 0,01 | 0,99 | 0,32 |
| **id** | **0,15** | **0,02** | **0,20** | **0,09** | **88,72** | **<0,001** |
| **researcher** | **0,32** | **0,32** | **0,42** | **0,25** | **268,19** | **<0,001** |
|  | Estimate | SE | df | Pr | Chisq | Pr |
| fixed effects | | | | | | |
| **feather length scaled** | **0,98** | **0,03** | **681,16** | **<0,001** |  | |
| morph | -0,15 | 0,06 | 232,32 | 0,02 |  |  |
| sex M | 0,12 | 0,09 | 126,88 | 0,20 |  |  |
| sex U | 0,40 | 0,30 | 131,39 | 0,18 |  |  |
| Year 2016 | -0,34 | 0,54 | 330,55 | 0,53 |  |  |
| Year 2017 | -0,52 | 0,55 | 329,39 | 0,34 |  |  |
| Year 2018 | -0,01 | 0,42 | 295,15 | 0,98 |  |  |
| **Year 2019** | **-1,12** | **0,45** | **294,18** | **0,01** |  |  |
| Year 2020 | -0,49 | 0,42 | 329,26 | 0,25 |  |  |
| Year 2021 | -0,60 | 0,39 | 294,81 | 0,13 |  |  |
| Year 2022 | -0,57 | 0,40 | 285,74 | 0,16 |  |  |
| Year 2023 | -0,53 | 0,40 | 327,67 | 0,19 |  |  |
| Year 2024 | -1,28 | 0,51 | 332,14 | 0,01 |  |  |

|  |  |  |  |  | LRT | |
| --- | --- | --- | --- | --- | --- | --- |
| **NB BARBS CHEST** | Var | SE | Prop V | SE | Chisq | Pr |
| random effects | | | | | | |
| residuals | 6,71 | 0,63 | 0,44 | 0,06 |  |  |
| **country** | **1,59** | **1,32** | **0,11** | **0,06** | **15,32** | **<0,001** |
| **id** | **6,80** | **1,03** | **0,45** | **0,06** | **64,13** | **<0,001** |
|  | Estimate | SE | df | Pr | Chisq | Pr |
| fixed effects | | | | | | |
| morph G | -0,06 | 0,42 | 226,91 | 0,89 |  | |
| sex M | -0,72 | 0,85 | 231,26 | 0,40 |  |  |
| sex U | 1,12 | 2,09 | 18,53 | 0,60 |  |  |
| Year 2016 | 0,14 | 3,22 | 219,05 | 0,97 |  |  |
| Year 2017 | -0,36 | 3,22 | 219,05 | 0,91 |  |  |
| Year 2018 | 1,93 | 2,68 | 219,62 | 0,47 |  |  |
| Year 2019 | 2,41 | 3,95 | 219,02 | 0,54 |  |  |
| Year 2020 | -0,01 | 2,61 | 223,46 | 1,00 |  |  |
| Year 2021 | 1,77 | 2,49 | 217,42 | 0,48 |  |  |
| Year 2022 | 2,42 | 2,61 | 212,37 | 0,35 |  |  |
| Year 2023 | 3,46 | 2,44 | 226,63 | 0,16 |  |  |
| Year 2024 | 2,63 | 3,00 | 224,14 | 0,38 |  |  |
| researcher SK | 0,83 | 1,21 | 18,16 | 0,50 |  |  |

|  |  |  |  |  |  |  |
| --- | --- | --- | --- | --- | --- | --- |
|  |  |  |  |  | LRT | |
| **NB BARBS BACK** | Var | SE | Prop V | SE | Chisq | Pr |
| random effects | | | | | | |
| residuals | 6,22 | 0,57 | 0,46 | 0,05 |  |  |
| country | 0,53 | 0,58 | 0,04 | 0,04 | 3,16 | 0,08 |
| **id** | **6,89** | **0,97** | **0,51** | **0,05** | **77,36** | **<0,001** |
|  | Estimate | SE | df | Pr | Chisq | Pr |
| fixed effects | | | | | | |
| morph | -0,40 | 0,41 | 255,18 | 0,33 |  | |
| sex M | 0,43 | 0,73 | 246,98 | 0,55 |  |  |
| sex U | 2,61 | 1,88 | 46,73 | 0,17 |  |  |
| Year 2016 | -2,01 | 3,19 | 235,92 | **0,53** |  |  |
| Year 2017 | -2,76 | 3,19 | 235,92 | 0,39 |  |  |
| Year 2018 | 0,00 | 2,57 | 233,35 | 1,00 |  |  |
| Year 2019 | -6,56 | 3,91 | 236,05 | 0,09 |  |  |
| Year 2020 | -0,85 | 2,52 | 241,59 | 0,74 |  |  |
| Year 2021 | -1,22 | 2,40 | 216,78 | 0,61 |  |  |
| Year 2022 | 0,08 | 2,43 | 218,74 | 0,97 |  |  |
| Year 2023 | 0,47 | 2,38 | 237,66 | 0,84 |  |  |
| Year 2024 | -1,52 | 3,02 | 238,69 | 0,62 |  |  |
| researcher SK | 2,84 | 0,84 | 13,48 | 0,00 |  |  |

|  |  |  |  |  | LRT | |
| --- | --- | --- | --- | --- | --- | --- |
| NB BARBULES CHEST | Var | SE | Prop V | SE | Chisq | Pr |
| random effects | | | | | | |
| residuals | 20,61 | 1,92 | 0,52 | 0,08 |  |  |
| **country** | **6,60** | **4,68** | **0,17** | **0,10** | **40,49** | **<0,001** |
| **id** | **12,07** | **2,36** | **0,31** | **0,06** | **31,68** | **<0,001** |
|  | Estimate | SE | df | Pr | Chisq | Pr |
| fixed effects | | | | | | |
| morph G | -0,53 | 0,64 | 223,55 | 0,40 |  | |
| **sex M** | **-2,89** | **1,23** | **222,01** | **0,02** |  |  |
| sex U | -4,21 | 3,63 | 13,48 | 0,27 |  |  |
| **researcher SK** | **4,46** | **4,78** | **215,24** | **0,35** |  |  |
| Year 2016 | 1,96 | 4,78 | 215,24 | 0,68 |  |  |
| Year 2017 | -1,04 | 4,02 | 223,07 | 0,80 |  |  |
| Year 2018 | 2,98 | 5,86 | 215,25 | 0,61 |  |  |
| Year 2019 | 1,02 | 3,89 | 220,66 | 0,79 |  |  |
| Year 2020 | 1,07 | 3,72 | 220,82 | 0,77 |  |  |
| Year 2021 | 2,49 | 3,88 | 221,80 | 0,52 |  |  |
| Year 2022 | 0,66 | 3,64 | 221,28 | 0,86 |  |  |
| Year 2023 | 3,06 | 4,48 | 220,51 | 0,50 |  |  |
| Year 2024 | 5,53 | 1,80 | 32,94 | 0,00 |  |  |

|  |  |  |  |  | LRT | |
| --- | --- | --- | --- | --- | --- | --- |
| NB BARBULES BACK | Var | SE | Prop V | SE | Chisq | Pr |
| random effects | | | | | | |
| residuals | 25,99 | 2,37 | 0,45 | 0,06 |  |  |
| **country** | **6,51** | **4,98** | **0,11** | **0,08** | **18,73** | **<0,001** |
| **id** | **25,51** | **3,79** | **0,44** | **0,06** | **64,45** | **<0,001** |
|  | Estimate | SE | df | Pr | Chisq | Pr |
| fixed effects | | | | | | |
| morph G | 0,12 | 0,83 | 243,77 | 0,88 |  | |
| sex M | 1,23 | 1,41 | 237,80 | 0,38 |  |  |
| sex U | 2,23 | 4,38 | 22,62 | 0,62 |  |  |
| **researcher SK** | **-0,43** | **6,26** | **230,29** | **0,95** |  |  |
| Year 2016 | -6,18 | 6,26 | 230,29 | 0,32 |  |  |
| Year 2017 | -3,14 | 5,12 | 236,06 | 0,54 |  |  |
| Year 2018 | -11,74 | 7,67 | 230,36 | 0,13 |  |  |
| Year 2019 | -2,08 | 4,99 | 236,71 | 0,68 |  |  |
| Year 2020 | -5,57 | 4,82 | 230,04 | 0,25 |  |  |
| Year 2021 | -2,30 | 4,87 | 231,95 | 0,64 |  |  |
| Year 2022 | -4,02 | 4,77 | 240,27 | 0,40 |  |  |
| Year 2023 | -6,51 | 6,01 | 237,36 | 0,28 |  |  |
| Year 2024 | 10,60 | 1,97 | 19,59 | 0,00 |  |  |

|  | Fam | Vic | Ned |
| --- | --- | --- | --- |
|  |  |  |  |
| 346 |  |  |  |
| 342 |  |  |  |
| 338 | SneD211 |  |  |
| 334 |  |  |  |
| 330 |  |  |  |
| 326 |  |  |  |
| 322 |  |  |  |
| 318 |  |  |  |
| 314 | Oe128 |  |  |
| 310 |  |  |  |
| 306 |  |  |  |
| 302 |  |  |  |
| 298 |  |  |  |
| 294 |  |  |  |
| 290 |  |  |  |
| 286 |  |  |  |
| 282 |  |  |  |
| 278 | Oe129 |  |  |
| 274 |  |  |  |
| 270 |  |  |  |
| 266 |  |  |  |
| 262 |  |  |  |
| 258 |  |  |  |
| 254 |  |  | Oe149 |
| 250 |  |  |  |
| 246 |  | Oe142 |  |
| 242 |  |  |  |
| 238 |  |  |  |
| 234 | SneD218 |  |  |
| 230 |  |  |  |
| 226 |  |  |  |
| 222 |  |  |  |
| 218 |  |  |  |
| 214 |  |  |  |
| 210 |  |  | SneD105 |
| 206 |  |  |  |
| 202 |  |  |  |
| 198 |  |  |  |
| 194 | SneD113 |  |  |
| 190 |  |  |  |
| 186 |  |  |  |
| 182 |  |  |  |
| 178 |  |  |  |
| 174 |  |  |  |
| 170 |  |  |  |
| 166 |  |  |  |
| 162 |  |  |  |
| 158 |  |  |  |
| 154 |  |  |  |
| 150 |  |  |  |
| 146 |  |  |  |
| 142 |  |  | So8G11 |
| 138 |  | So15A6 |  |
| 134 |  |  |  |
| 130 | So1C6 |  |  |
| 126 |  |  |  |
| 122 |  |  |  |
| 118 |  |  |  |
| 114 |  |  |  |
| 110 |  |  |  |
| 106 |  |  |  |
| 102 |  |  |  |
