# Supplementary material for: Dressed for the Weather: Tawny Owl Feather Adaptations Across a Climatic Gradient
Source: Ecol Evol. 2025 Jun 24;15(6):e71441. doi: 10.1002/ece3.71441 (PMC12185932; doi:10.1002/ece3.71441)
Supplement: Supplementary file 2 — Figure S1. [file ECE3-15-e71441-s001.docx]

|  | Fam | Vic | Ned |
| --- | --- | --- | --- |
|  |  |  |  |
| 346 |  |  |  |
| 342 |  |  |  |
| 338 | SneD211 |  |  |
| 334 |  |  |  |
| 330 |  |  |  |
| 326 |  |  |  |
| 322 |  |  |  |
| 318 |  |  |  |
| 314 | Oe128 |  |  |
| 310 |  |  |  |
| 306 |  |  |  |
| 302 |  |  |  |
| 298 |  |  |  |
| 294 |  |  |  |
| 290 |  |  |  |
| 286 |  |  |  |
| 282 |  |  |  |
| 278 | Oe129 |  |  |
| 274 |  |  |  |
| 270 |  |  |  |
| 266 |  |  |  |
| 262 |  |  |  |
| 258 |  |  |  |
| 254 |  |  | Oe149 |
| 250 |  |  |  |
| 246 |  | Oe142 |  |
| 242 |  |  |  |
| 238 |  |  |  |
| 234 | SneD218 |  |  |
| 230 |  |  |  |
| 226 |  |  |  |
| 222 |  |  |  |
| 218 |  |  |  |
| 214 |  |  |  |
| 210 |  |  | SneD105 |
| 206 |  |  |  |
| 202 |  |  |  |
| 198 |  |  |  |
| 194 | SneD113 |  |  |
| 190 |  |  |  |
| 186 |  |  |  |
| 182 |  |  |  |
| 178 |  |  |  |
| 174 |  |  |  |
| 170 |  |  |  |
| 166 |  |  |  |
| 162 |  |  |  |
| 158 |  |  |  |
| 154 |  |  |  |
| 150 |  |  |  |
| 146 |  |  |  |
| 142 |  |  | So8G11 |
| 138 |  | So15A6 |  |
| 134 |  |  |  |
| 130 | So1C6 |  |  |
| 126 |  |  |  |
| 122 |  |  |  |
| 118 |  |  |  |
| 114 |  |  |  |
| 110 |  |  |  |
| 106 |  |  |  |
| 102 |  |  |  |

Figure S1: microsatellite peak profiles for microsatellite genotyping
